# Bioinformatics Analysis Reveals FOXM1/BUB1B Signaling Pathway as a Key Target of Neosetophomone B in Human Leukemic Cells: A Gene Network-Based Microarray Analysis

**DOI:** 10.3389/fonc.2022.929996

**Published:** 2022-07-01

**Authors:** Shilpa Kuttikrishnan, Tariq Masoodi, Gulab Sher, Ajaz A. Bhat, Kalyani Patil, Tamam El-Elimat, Nicholas H. Oberlies, Cedric J. Pearce, Mohmmad Haris, Aamir Ahmad, Feras Q. Alali, Shahab Uddin

**Affiliations:** ^1^ Translational Research Institute, Academic Health System, Hamad Medical Corporation, Doha, Qatar; ^2^ College of Pharmacy, Qatar University, Doha, Qatar; ^3^ Laboratory of Molecular and Metabolic Imaging, Cancer Research Department, Sidra Medicine, Doha, Qatar; ^4^ Department of Medicinal Chemistry and Pharmacognosy, Faculty of Pharmacy, Jordan University of Science and Technology, Irbid, Jordan; ^5^ Department of Chemistry and Biochemistry, University of North Carolina at Greensboro, Greensboro, NC, United States; ^6^ Mycosynthetix, Inc., Hillsborough, NC, United States; ^7^ Laboratory of Animal Research Center, Qatar University, Doha, Qatar; ^8^ Dermatology Institute, Academic Health System, Hamad Medical Corporation, Doha, Qatar

**Keywords:** fungal metabolites, Neosetophomone B, FOXM1, BUB1B, apoptosis, leukemia, cell-cycle checkpoints, TCGA

## Abstract

Abnormal expression of Forkhead box protein M1 (FOXM1) and serine/threonine kinase Budding uninhibited by benzimidazoles 1 (BUB1B) contributes to the development and progression of several cancers, including chronic myelogenous leukemia (CML). However, the molecular mechanism of the FOXM1/BUB1B regulatory network and the role of Neosetophomone-B (NSP-B) in leukemia remains unclear. NSP-B, a meroterpenoid fungal secondary metabolite, possesses anticancer potential in human leukemic cells lines; however, the underlying mechanism has not been elucidated. The present study aimed to explore the role of NSP-B on FOXM1/BUB1B signaling and the underlying molecular mechanism of apoptosis induction in leukemic cells. We performed gene expression profiling of NSP-B-treated and untreated leukemic cells to search for differentially expressed genes (DEGs). Interestingly *BUB1B* was found to be significantly downregulated (logFC -2.60, adjusted p = 0.001) in the treated cell line with the highest connectivity score among cancer genes. Analysis of TCGA data revealed overexpression of *BUB1B* compared to normal in most cancers and overexpression was associated with poor prognosis. *BUB1B* also showed a highly significant positive correlation with *FOXM1* in all the TCGA cancer types. We used human leukemic cell lines (K562 and U937) as an *in vitro* study model to validate our findings. We found that NSP-B treatment of leukemic cells suppressed the expression of FOXM1 and BUB1B in a dose-dependent manner. In addition, NSP-B also resulted in the downregulation of FOXM1-regulated genes such as Aurora kinase A, Aurora kinase B, CDK4, and CDK6. Suppression of FOXM1 either by siRNA or NSP-B reduced BUB1B expression and enhanced cell survival inhibition and induction of apoptosis. Interestingly combination treatment of thiostrepton and NSP-B suppressed of cell viability and inducted apoptosis in leukemic cells *via* enhancing the activation of caspase-3 and caspase-8 compared with single-agent treatment. These results demonstrate the important role of the FOXM1/BUB1B pathway in leukemia and thus a potential therapeutic target.

## Introduction

Leukemia is a type of blood cancer characterized by the uncontrolled proliferation and lack of proper differentiation of hematopoietic cells causing the accumulation of non-functional leukocytes and their progenitors, primarily in the bone marrow and lymphatic system (1). According to GLOBOCAN 2020 statistics, leukemia was the 11th leading cause of cancer-related mortality worldwide (1). Leukemia accounted for approximately 3.4% (474,519) of all new cancer cases and 3.8% (311,549) of all cancer deaths in 2020, representing 2.5% of all cancer sites/types reported (2). The most common type of leukemia, acute myeloid leukemia (AML), has a high mortality rate and is difficult to treat (3). Currently, the mainstay treatment of AML includes chemotherapy, radiotherapy, and bone marrow transplantation. Even though these therapies have increased patient survival rates, some patients develop resistance and relapse (4). As a result, finding new strategies to treat leukemia with minimal side effects remains a significant therapeutic problem. Recently, natural compounds have gained considerable interest as abundant and emerging sources for developing novel anticancer therapies due to improved efficacy and reduced side effects (5). In recent years combinational therapeutic regimen involving the use of chemotherapeutic drugs and natural compounds is now considered a new innovative approach for overcoming multidrug resistance and normal cell toxicity (6).

Natural products and their derivatives, endowed with structural diversity and a range of pharmacological and molecular properties have shown great promise in the development of cancer therapies (7). Neosetophomone B (NSP-B), a meroterpenoid fungal secondary metabolite, isolated from a *Neosetophoma sp*. has been reported to be cytotoxic even at micromolar concentrations in breast and ovarian cancer cell lines (8). Recently NSP-B has been shown to cause cell death in leukemic cells *via* inhibition of AKT/SKP2 axis and activation of mitochondrial and caspase signaling cascades (9).

Budding uninhibited by benzimidazoles 1 (BUB1B), a mitotic checkpoint serine/threonine kinase, that serves an important role in chromosome alignment, has been shown to act as a tumor promoter in many cancers (10–12). It has been shown that complete deletion of BUB1B in the mouse germline causes early embryonic mortality (13). Furthermore, lowering BUB1B levels or inhibiting BUB1B kinase activity in human cancer cells causes significant chromosomal loss and apoptotic cell death (14). Knockdown of BUB1B has been shown to reduce tumor growth *in vivo* (15). It has been demonstrated that Forkhead box protein M1 (FOXM1) regulates BUB1B expression through transcriptional regulation by binding to and activating the BUB1B promoter (16). FOXM1 is a transcription factor also known as a master regulator of tumor metastasis and has been reported to regulate a wide range of biological activities, including cell proliferation, cell cycle progression, cell differentiation, DNA damage repair, tissue homeostasis, angiogenesis, and apoptosis, among others (17, 18). Gene silencing of FOXM1 or suppression of its expression with siomycin A reduced BUB1B expression and decreased cell growth (15). This study suggests that the crosstalk between FOXM1 and BUB1B plays an essential role in the growth and survival of cancer cells. Co-targeting these signaling pathways may be a viable strategy to induce cancer cell death.

In our recent study, we explored the effect of NSP-B on cell proliferation, cell cycle, and apoptosis in leukemic cells (9). In the present study, we performed bioinformatic analysis to screen the targets of NSP-B in leukemic cells. Furthermore, we validated these screened targets with gene profiles of leukemia patients, to understand the underlying mechanism of pathogenesis of leukemia.

## Materials and Methods

### Reagents and Antibodies

Cell Counting Kit-8(CCK-8), methanol, dimethylsulfoxide (DMSO),thiostrepton were obtained from Sigma-Aldrich (St. Louis, MO, USA). Antibodies against FOXM1, Aurora Kinase A,B,CDK-6,-4, PARP, cleaved caspase-8,-3, and β-actin were obtained from Cell Signaling Technologies (Beverly, MA, USA).BUB1B, HSP60, p-H2AX, Bax, Bcl2 were obtained from Santa Cruz Biotechnology, Inc., (CA, USA). AllPrep DNA/RNA Mini Kit was purchased from Qiagen (Hilden, Germany). Live and Dead assay kit, RPMI 1640 medium, fetal bovine serum (FBS), penicillin and streptomycin were obtained from Life Technologies, Inc. (Carlsbad, CA).

### Cell Culture and The Natural Compound NSP-B

Leukemic cell lines K562 and U937 were purchased from ATCC (Manassas, Virginia, USA), and maintained in RPMI 1640 medium supplemented with 10% fetal bovine serum,100 U/ml penicillin, and 100 U/ml streptomycin at 37°C in humidified incubator comprising of 5% CO_2_ (19).The natural compound NSP-B was isolated from the fungal strain *Neosetophoma* sp. [strain MSX50044], as mentioned earlier (8).

### Expression Profiling

We investigated gene expression in NSP-B treated K562 cells by comprehensive transcriptome analysis using the high-resolution Human Transcriptome Array 2.0 (HTA 2.0) (Applied Biosystems™) containing >6.0 million distinct probes covering >285,000 transcripts. K562 cells were treated with NSP-B (10 μM) in duplicate for 48 hours. Total RNA was extracted from the cell lines using AllPrep DNA/RNA Mini Kit (Hilden, Germany). The quantity and quality of the RNA was checked by NanoDrop^®;^ Spectrophotometer (Thermo Scientific™), Qubit RNA HS Assay Kit (Invitrogen™), and Qubit RNA IQ Assay Kit (Invitrogen™). GeneChip™ WT PLUS Reagent Kit (Applied Biosystems™) was used to prepare samples for hybridization on the HTA 2.0 arrays. In brief, 100 ng of total RNA was reverse transcribed to cDNA. cRNA was prepared from the cDNA and purified with purification beads. ss-cDNA was synthesized from the cRNA and purified by the beads. The ss-cDNA was fragmented, labeled, and added to the hybridization master mix to prepare a hybridization cocktail. 200 µL of the cocktail was added to the probe array cartridge and incubated with rotation at 60 rpm for 16 hours at 45°C in the hybridization oven. GeneChip™ Hybridization, Wash and Stain Kit (Applied Biosystems™) was used to process the arrays on the Fluidics Station 450. Finally, the arrays were scanned with the Scanner 3000 7G to generate CEL files (raw data).

### Dysregulated Genes, Network, and Pathway Analysis

Treated and untreated CEL files in duplicates were processed and analyzed by different packages in R version 4.1.1 (https://www.R-project.org). Quality control (QC) metrics were generated and differentially expressed genes (DEGs) between untreated and treated cell lines were analyzed using limma R package (20). Log2 values with a fold change (FC) ≥ 1.5 and ≤ -1.5 with p < 0.05 were used to identify upregulated and downregulated genes, respectively. Statistically significant DEGs were inputted to Ingenuity Pathway Analysis (IPA) for the identification of key activated and inhibited signaling pathways, gene networks, molecular and cellular biological functions. Further, activated or inhibited upstream regulators were identified with an inactivation or inhibition score of ±2 using IPA. Protein-protein interaction (PPI) analysis of DEGs was performed using STRING tool (21) and results were visualized by Cytoscape. An interaction score of ≥0.4 was used as the cut-off for the network and all the interactions below the cut-off were termed as weak and removed. Genes in the PPI network having a connectivity score >20 was referred as potential hub genes.

The PPI interactions from the STRING were used as input to Cytoscape version v3.9.0 to visualize the interactions and identify the hub genes (22). High confidence clusters were identified by setting kappa score (K-score) to 5, degree to 2, maximum depth to 100, and node score to 0.2 in the network (23). In order to find biological relevance of hub genes, cancer gene annotation was performed, and key tumor suppressors and oncogenes were identified.

### Expression and Correlation of Hub Genes

Expression data of key hub genes was downloaded from Genomic Data Commons (GDC) of The Cancer Genome Atlas (TCGA) for AML and Lymphoid Neoplasm Diffuse Large B-cell Lymphoma (DLBC). Expressed data was further supplemented with clinical data of the patients to perform clinical associations for diagnosis and prognosis. In order to identify importance of cancer hub genes, we extended our analysis to include the entire solid tumor in TCGA and differential expression analysis was performed. Further, Spearman correlation analysis was carried out to identify key co-expressing genes with our gene of interest.

### Prognosis of Key Hub Genes

To derive biological relevance, clinical associations of cancer hub genes was performed using clinical and expression data from TCGA. ANOVA and Mann–Whitney U test were applied to see the differences between different clinical subtypes and groups wherever appropriate. Survival curves were obtained using the survival package in R by dividing the patients into low and high expression groups. The log-rank p-value and hazard ratio (HR) with 95% confidence interval (CI) were obtained for clinical interpretations.

### Cell Viability Assay

Leukemic cell lines K562 and U937 were treated in the presence and absence of NSP-B and thiostrepton, and the cell viability was measured using CCK-8 colorimetric method as mentioned earlier (19).

### Live/Dead Assay

Leukemic cells K562 and U937 were treated with various doses of NSP-B and thiostrepton and the stain was prepared according to the protocol. The cells were stained and visualized by EVOS FLoid Cell Imaging System from Invitrogen (Thermo Fisher Scientific) (19).

### Cell Lysis and Immunoblotting

Leukemic cells K562 and U937 were treated with NSP-B and thiostrepton and the cells were lysed as described previously (19).An equal amount of protein was separated by SDS-page, transferred into the PVDF membrane, and then immunoblotted with various antibodies and visualized.

### Gene Silencing

Leukemic cells K562 were transfected with FOXM1 siRNA (Cat no: SI00421050, Qiagen, Germany) and Control siRNA (Cat no: 1027281, Qiagen) using SF Cell Line-4D-Nucleofector™ System (Lonza) as per the protocol. After incubation, cells were harvested, lysed and probed with anti- FOXM1 and other different antibodies (19).

### Synergism Analyses

Synergy was quantified using the Chou–Talalay method using the CalcuSyn software to calculate the values of the combination index (http://www.biosoft.com/w/calcusyn.htm, Biosoft) (24). The dose–effect curve for each drug alone is determined on the basis of experimental observations using the median-effect principle and is compared with the effect achieved with a combination of the two drugs to derive a combination index (CI) value. The CI indicates the level of synergism or antagonism: <0.9 indicates synergism (0.3–0.7 strong; 0.7–0.85 moderate; 0.85–0.9 slight), 0.9–1.1 nearly additive effect, and >1.1 antagonism (25).

### Statistical Analysis

All statistical analyses were performed in R version 4.1.1. The Mann–Whitney U Test, the Chi-Squared Test or Fisher’s Exact Test were executed to compare the continuous and categorical variables. Correlation analysis was performed using Spearman’s rank correlation method. All the statistical tests were two-tailed with p < 0.05 considered statistically significant. For differential expression of genes, the p-values were adjusted with false discovery rate (FDR) of <10%.

## Results

### Differentially Expressed Genes and Pathways

**Figure 1:**
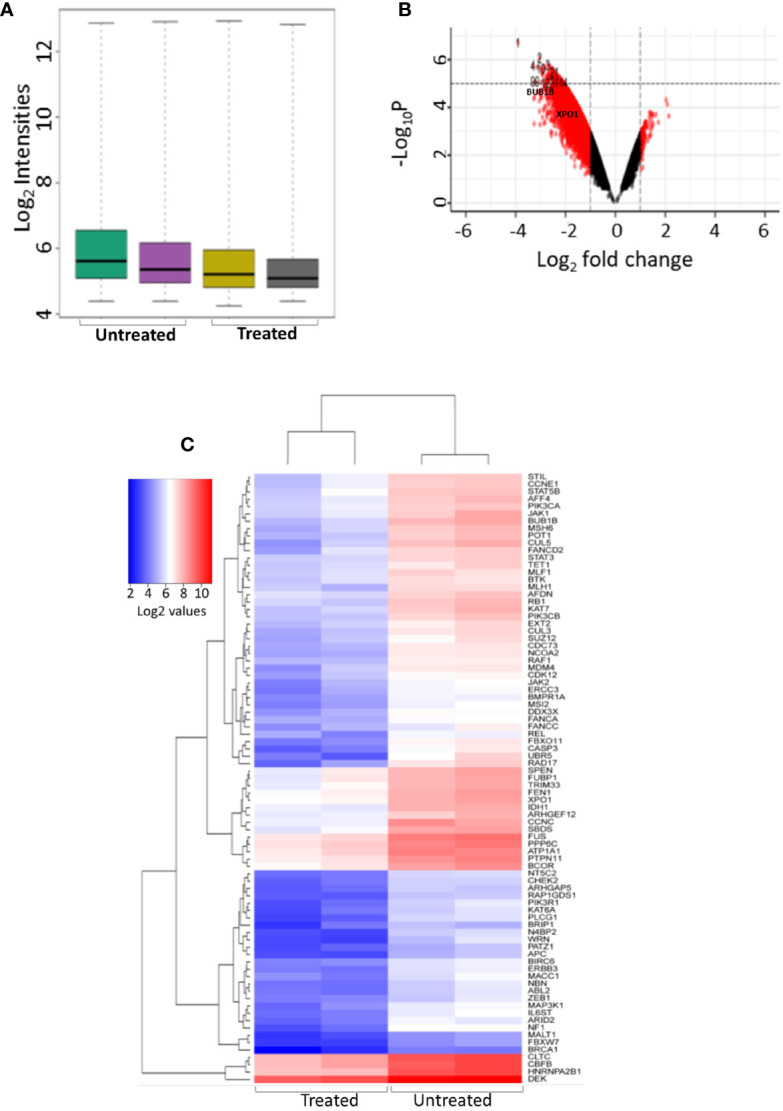
Differential gene expression of treated and untreated K562 cell line using microarray. **(A)** Intensity plot displaying the quality of the microarray CELL data **(B)** Volcano plot showing the differentially expressed genes between K562 cell line treated with NSP-B (10µM) and untreated K652 cells, A log_2_ fold change (FC) cutoff of ± 1.5 was used for significant differentially expressed genes with p < 0.05. **(C)** Heatmap displaying the expression of significant differentially expressed cancer genes between treated and untreated K562 cells.

**Figure 2:**
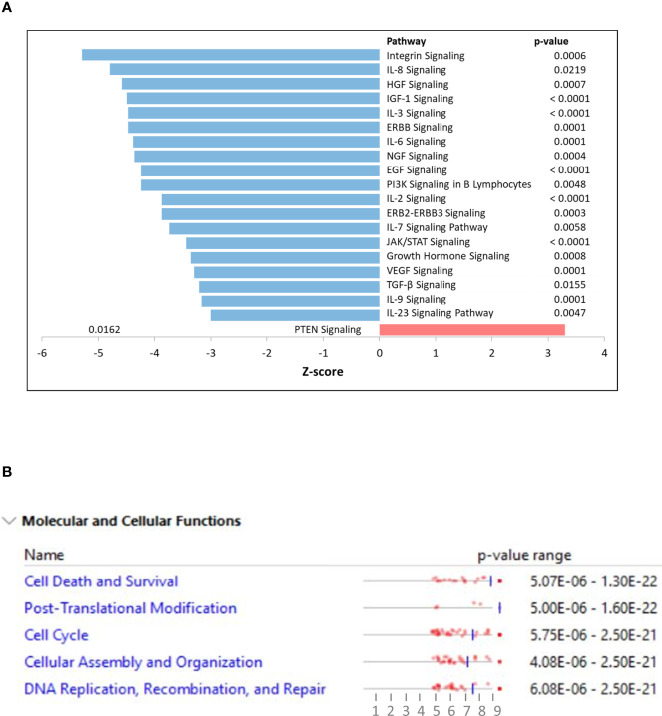
**(A)** Ingenuity pathway analysis (IPA) displaying top affected signaling pathways with their z-scores. Positive z-score is predicted to be activated whereas negative z-score as inhibition of the pathway. **(B)** Ingenuity pathway analysis (IPA) displaying top affected molecular and cellular functions using differentially expressed genes as input.

K562 cells were treated with NSP-B (10μM) in duplicate for 48 h, and transcript expressions were analyzed using the HTA 2.0 array containing >6.0 million distinct probes covering >85,000 transcripts. The data was analyzed using different R packages, including ArrayExpress, Oligo, arrayQualityMetrics, limma and ggplot2. Quality metrics of the data was obtained, data preprocessed and DEGs were identified (**Figure 1A**). For identifying DEGs, the logFC of ≥ 1.5 and ≤ -1.5 with statistically significant p value (p < 0.05) were used for up-regulated and down-regulated genes, respectively. The p values were adjusted with FDR of 10%. Overall, 2,079 significant DEGs were identified with p < 0.05 in the NSP-B treated cell line. This included 2,072 down-regulated genes and only 7 genes were found to be up-regulated (**Supplementary Table 1**). The volcano plot and heatmap of the expression data were obtained with heatmap restricted to only cancer genes (**Figures 1B, C**). Genes with logFC ≥ 1.5 and ≤ -1.5 were submitted to IPA to identify pathways and gene ontologies (GO) associated with DEGs. The highly significant pathways associated with treatment were inhibition of ERBB Signaling, EGF Signaling, PI3K Signaling, JAK/STAT Signaling, VEGF Signaling, TGF-β Signaling and likely activation of PTEN Signaling. (**Figure 2A**). The significant GOs found in molecular and cellular functions category were inhibition of cell cycle progression and DNA replication (**Figure 2B**).

### PPI Network Analysis and Functional Annotations of the Selected Potential Targets

**Figure 3:**
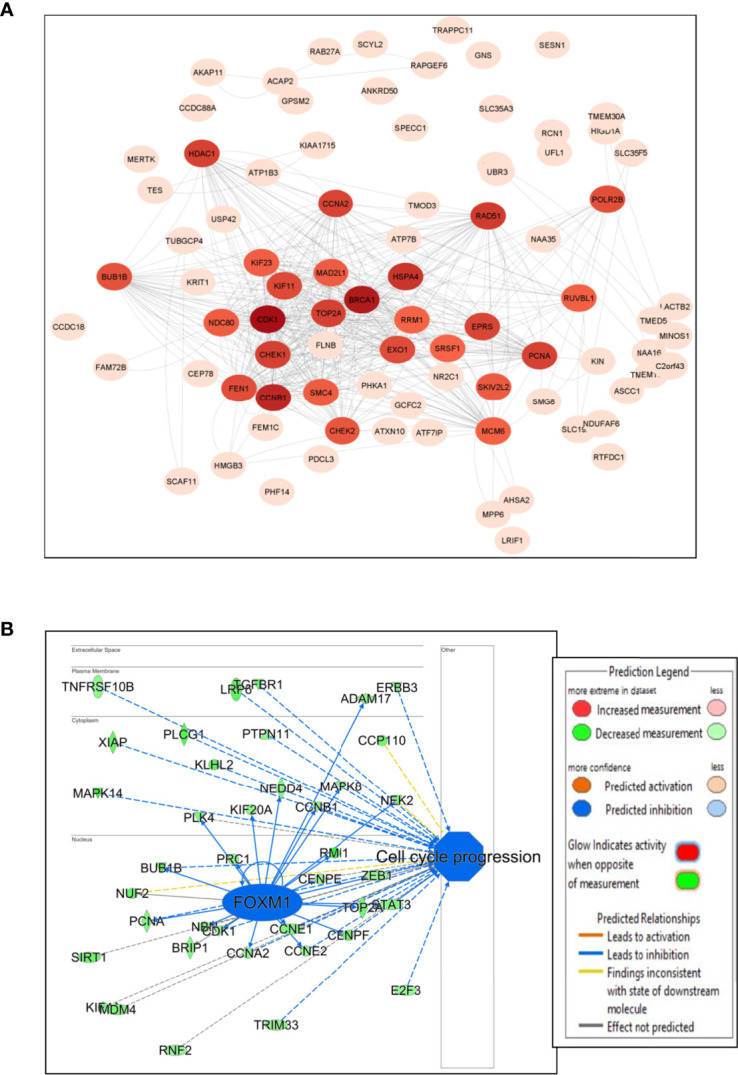
Network and pathway analysis. **(A)** Protein-protein interaction network using Cytoscape. The color intensity of the circles with genes depends on the number of connections with intense color (hub genes) having large number of connections and light color with less connections. **(B)** IPA transcriptional analysis of *FOXM1* and its interaction and connectivity with other differentially expressed genes in the network. The predicted inhibition of *FOXM1* likely inhibits cell cycle progression.

The PPI network was constructed for the DEGs using STRING with a confidence interaction score of ≥ 0.4. The interaction scores were analyzed and visualized in Cytoscape to identify hub genes and clusters (**Figure 3A**). We found approximately 37 hub genes having a degree value of over 200 including down-regulation of two oncogenes (*BUB1B* and *XPO1)*, with *BUB1B* comparatively having a higher number of connections (**Supplementary Table 1**). The hub genes are displayed as darker color in the figure, and color intensity depends on the number of connections (**Figure 3A**). *FOXM1* regulates *BUB1B* expression through transcriptional regulation by binding to and activating the BUB1B promoter (15). IPA analysis showed many targets of *FOXM1* are down-regulated (green color), which likely inhibits *FOXM1* (blue color; predicted inhibition by IPA), thus likely inhibiting cell cycle progression (**Figure 3B**).

### Co-Expression and Clinical Correlation of Selected Genes

**Figure 4:**
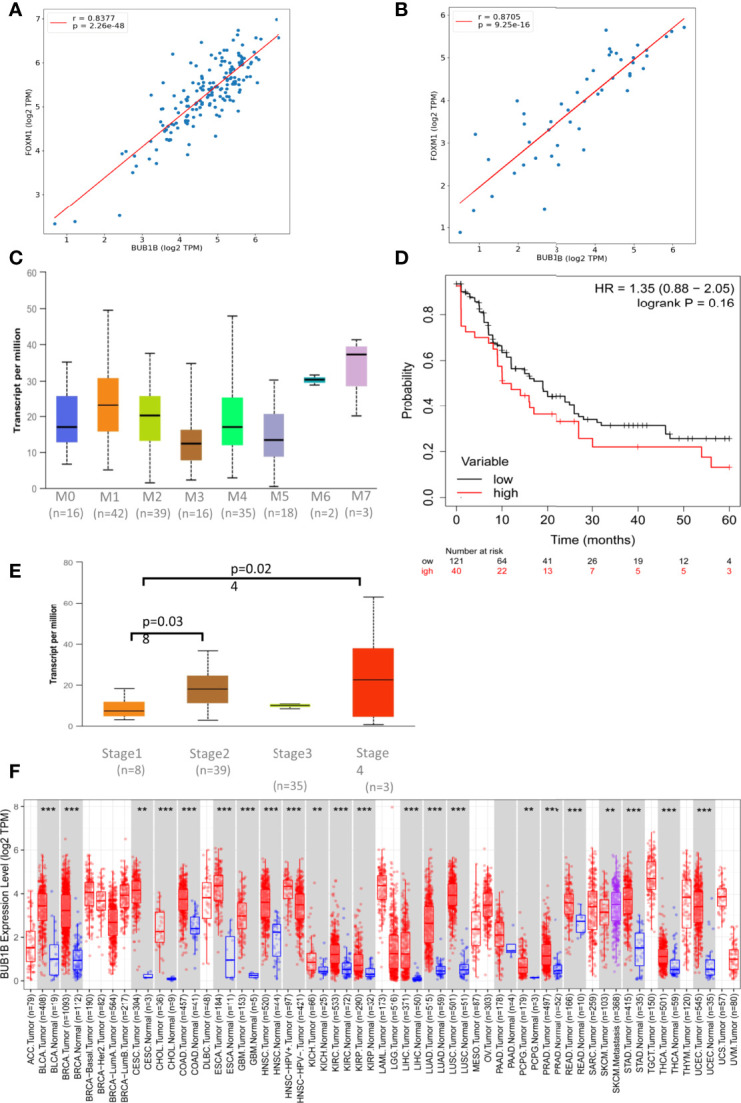
*BUB1B* expression and association in TCGA: **(A)** Spearman correlation between *BUB1B* and *FOXM1* in AML plotted using log2 Transcript Count Per Million (TPM) expression values. **(B)** Spearman correlation between *BUB1B* and *FOXM1* in DLBC plotted using log2 TPM expression values. **(C)** Expression of *BUB1B* in different subtypes of Acute Myeloid Leukemia (AML). The expression values are normalized in TPM. **(D)** Kaplan Meier Curve showing overall survival between *BUB1B* high and low expression patients in AML. **(E)** Expression of *BUB1B* in different stages of DLBC. The expression values are normalized and plotted as Log2 TPM. **(F)** Log2 TPM expression of *BUB1B* in TCGA hematological and solid malignancies. Significant differential expression between tumor and normal tissues or between tumor subtypes is calculated using Wilcoxon test and the significance level is annotated by the number of stars on top of box plots (**p < 0.01; ***p < 0.001). This figure was generated using TIMER2.0 (http://timer.cistrome.org/) TCGA abbreviations are expanded in **Supplementary Figure 1**.

We investigated the co-expression of *BUB1B* with *FOXM1* using expression data from TCGA. *BUB1B* is positively correlated with *FOXM1* in all the cancer types from TCGA. It showed highly significant positive correlation in AML (spearman r = 0.8377; p = 2.26e-48) (**Figure 4A**) and Lymphoid Neoplasm DLBC (spearman r = 0.8705; p = 9.25e-16) (**Figure 4B**). We further extended this to other solid tumors in TCGA, and interestingly a highly significant correlation was observed between *BUB1B* and *FOXM1* (**Supplementary Figure 1**).

To see if the high expression of *BUB1B* has any prognostic importance, we investigated its clinical association and overall survival in AML and DLBC from TCGA. We found high expression of *BUB1B* in M6 and M7 subtypes of AML, however the association was not statistically significant due to small number of samples (**Figure 4C**). The median overall survival for AML patients with high BUB1B expression was ~10 months in comparision to ~19 months for patients exhibiting low expression of BUB1B; however the association was not statistically significant (HR 1.35, CI 0.88-2.05; logrank p=0.16) (**Figure 4D**). In DLBC, high expression of *BUB1B* was found to be associated with higher tumor stage (Stage1 vs 2, p=0.038; Stage1 vs 4, p=0.024. **Figure 4E**). Overall survival was not calculated in DLBC due to fewer patients and survival events. Further, *BUB1B* was found to be significantly overexpressed in most TCGA tumor types (**Figure 4F**). We performed overall survival analysis of *BUB1B* in other solid tumors in TCGA and found that high expression of *BUB1B* is associated with poor overall survival in six different cancer types (**Supplementary Figure 2**).

### Effect of NSP-B on FOXM1, BUB1B and Downstream Key Targets: Validation of Microarray Data

**Figure 5:**
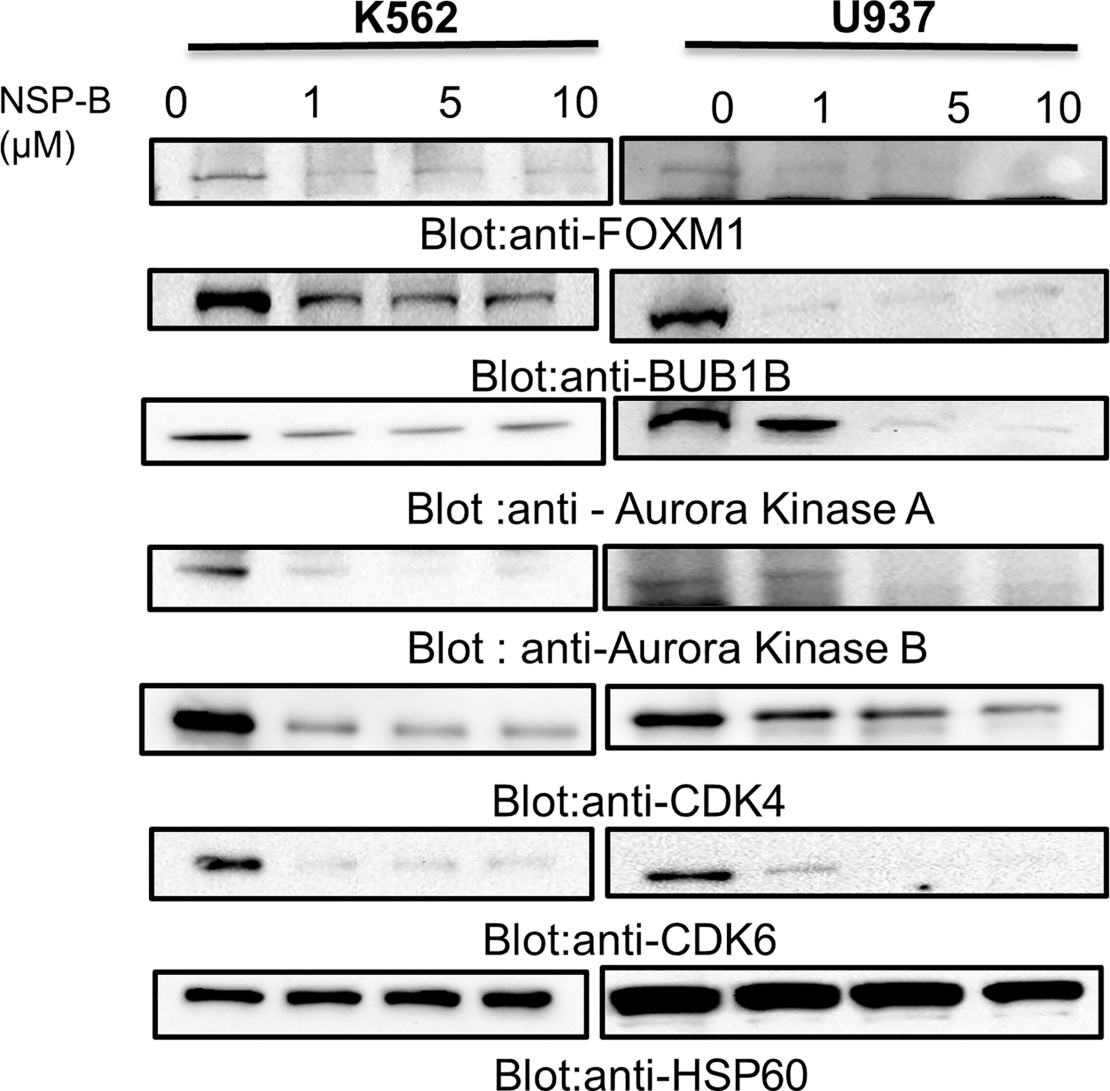
NSP-B mediated downregulation of FOXM1 and its downstream targets in leukemic cells. K562 and U937 cells were treated with increasing doses of NSP-B and equal amount of proteins were immunoblotted against various antibodies such as FOXM1, BUB1B,Aurora Kinases A and B, CDK 4 and 6 and HSP60.NSP-B treatment caused the downregulation of FOXM1 and its downstream targets BUB1B, Aurora Kinases A and B, CDKs 4 and 6.

To substantiate the results of the microarray studies, immunoblotting was performed to assess the protein expression of FOXM1, BUB1B and downstream targets such as Aurora kinase A, Aurora kinase AB, CDK4, and CDK6. We found that NSP-B treatment of K562 and U937 cells inhibited the expression of FOXM1 and BUB1B in a dose dependent manner confirming the reliability of our microarray results at the protein level (**Figure 5**). In addition, NSP-B also suppressed the expression of Aurora kinase A, Aurora kinase B, CDK4 and CDK6 (**Figure 5**).

### Knockdown of FOXM1 Resulted in Growth Inhibition, Suppression of BUB1B and Activation of Caspase-Cascade

**Figure 6:**
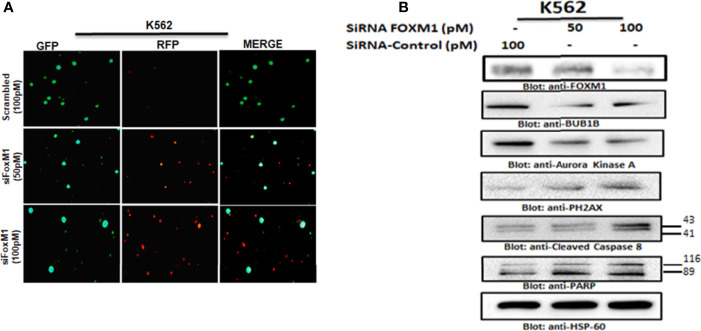
Gene silencing of FOXM1 suppressed BUB1B expression. **(A)** K562 cells were transfected with control (100 pM) and FOXM1 siRNA (50 and 100 pM) as indicated. After 48 hours of transfection Live and dead assay was performed for detection of live and dead cells after FOXM1 knockdown. **(B)** K562 cells were transfected with control (100 pM) and FOXM1 siRNA (50 and 100 pM) as indicated in Materials and Methods. Immunoblot analysis of K562 cells transfected with control (100 pM) and FOXM1 siRNA (50 and 100 pM). Cells were lysed, and an equal amount of proteins for each sample were loaded onto the SDS-polyacrylamide gel. Membranes were blotted against against FOXM1, BUB1B, Aurora Kinase A, p-H2AX, Cleaved caspase-8, PARP and HSP60.

To investigate that FOXM1 regulates cell proliferation and BUB1B expression in leukemic cells, we silenced the FOXM1 expression in K562 cells using specific siRNA against FOXM1. As shown in **Figure 6A**, the siRNA knockdown of FOXM1 resulted in the increased level of red floresence,which is a measure of dead cells. Immunoblotting data furher showed that gene silencing of FOXM1 in leukemic cells down regulated the expression of BUB1B as well as Aurora kinase A, a downstream target of FOXM1 (**Figure 6B**). Interestingly, apoptotic markers including p-H2AX, caspase-8, and PARP were also found to be activated after FOXM1 knockdown (**Figure 6B**). These results suggest that the knockdown of FOXM1 inhbits cell growth and triggers apoptosis *via* supression of BUB1B in leukemic cells.

### Synergistic Activity Between Thiostrepton and NSP-B in Leukemic Cells

As we showed that NSP-B inhibits FOXM1 expression in leukemic cells cells, next we wanted to investigate if thiostrepton, a specific and direct inhibitor of the FOXM1 protein, could act synergistically with NSP-B in inhibiting cell proliferation and inducing apoptosis. To determine the combination dose of thiostrepton and NSP-B that posseses maximal cytotoxic effects, K562 and U937 cells were treated with various combinations of thiostrepton and NSP-B and cell viability was determined. The combination index (CI) values of sub toxic doses of thiostrepton and NSP-B were calculated using Calcusyn software of Chou- Talalay method (26, 27). CI represents the quantitative interaction between drugs and CI values < 1 (synergy), = 1 (additive effect), and >1 (antagonism), as explained by Chou and Talalay (27). The synergistic effects of GS and thiostrepton on cell viability in K562 and U937 cell lines were found at 1 µM of GS and 1 µM of thiostrepton with a CI index of 0.328 and 0.437 respectively. (**Supplementary Tables 2, 3** **)** (**Supplementary Figures 3A, B**).

### Co-Treatment With Thiostrepton and NSP-B Augmented Inhibition of Cell Viability and Induced Apoptosis *Via* Mitochondrial Apoptotic Pathway in Leukemic Cells

**Figure 7:**
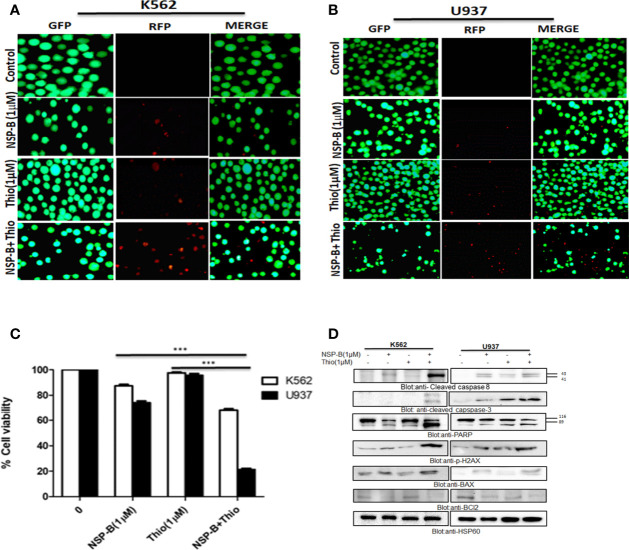
Cotreatment of NSP-B and thiostrepton augmented the inhibition of cell viability and induces apoptosis in leukemic cells. **(A)** K562 and **(B)** U937 were treated with NSP-B and thiostrepton alone and in combination for 48 hours. After incubation the cells were stained with live and dead reagent and visualized under a fluorescent microscope. **(C)** K562 and U937 cells cells were treated with 1µM of NSP-B and 1µM of thiostrepton for 48 hours and cell viability assay was performed as mentioned in Materials and Methods. The graph displays the mean ± SD (standard deviation) of three independent experiments. ***p < 0.001. **(D)** K562 and U937 were treated with 1 µM of NSP-B and thiostrepton alone and in combination and cells were lysed and separated using SDS-PAGE, transferred to a PVDF membrane, and immunoblotted with antibodies such as PARP, cleaved caspase-8, cleaved caspase-3, BAX, Bcl-2, P-H2AX, and HSP60.

Using the Chou-Talalay isobologram equation, we optimized the doses of thiostrepton and NSP-B at 1 µM each for maximal synergistic effects. We performed several experiments in K562 and U937 cells to assess the combination effects of thiostrepton and NSP-B, as compared with single agent treatments. We first performed a live and dead cell assay using LIVE/DEAD*^®;^* Cell Imaging Kit as described in Materials and Methods. We found that the combination of thiostrepton and NSP-B in K562 and U937 cells triggered cell death to a greater extent than individual treatments (**Figures 7A, B**). Next CCK-8 assay was performed in K562 and U937 cells to determine the number of viable cells upon treatment. We found that the combination of thiostrepton and NSP-B siginificatly (p<0.001) inhibited the proliferation of K562 and U937 cells compared to single agent treatment, as shown in **Figure 7C**. The results demonstrated that co-treatment with thiostrepton and NSP-B exerts synergistic effects on the inhibition of cell viability in leukemic cells. To further examine whether the observed suppression of cell viability involved apoptosis, K562 and U937 cells were treated with 1 μM of thiostrepton or 1μM of NSP-B or both, for 24 *h* prior to the determination of caspase-3 and caspase-8 activities. As shown in **Figure 7D**, the combination of thiostrepton and NSP-B showed a significant increase in the activation of capase 8, caspase3 and PARP as compared to individual treatments. Combination of thiostrepton and NSP-B also increased expression levels of p-H2AX, a marker of double strand breaks (**Figure 7D**). Finally, cotreated cells showed an increase in the expression levels of Bax and a decrease in the Bcl-2 levels leading to an increased ratio of Bax/Bcl2 in leukemic cells suggesting mitochondrial mediated apoptosis. These results indicate the synergistic cytotoxicity of thiostrepton and NSP-B, and that the apoptotic mechanism was caspase-3/-8-dependent in leukemic cells.

## Discussion

Natural compounds have increasingly become important as anticancer agents against several cancers. A number of bioactive natural substances have been demonstrated to be effective in preventing and treating cancer *via* targeting diverse signaling molecules and pathways (28, 29). In addition, increasing evidence suggests that many naturally occurring compounds improve the efficacy of chemotherapy (7). NSP-B, a meroterpenoid fungal secondary metabolite, isolated from an undescribed *Neosetophoma sp* (8) has recently been shown to induce cytotoxicity *via* triggering apoptosis in leukemic cell lines (9). These encouraging results prompted us to investigate the more detailed anti-proliferative and proapoptotic mechanism of NSP-B in leukemic cells. In the present study, we screened and verified several novel and promising NSP-B target genes in leukemia cells *via* bioinformatics analysis approach. We investigated gene expression in NSP-B treated K562 cells by comprehensive transcriptome analysis using the high-resolution Human Transcriptome Array 2.0 (HTA 2.0). Pathway analysis of DEGs using IPA revealed inhibition (z-score < -2.0) of ERBB Signaling, EGF Signaling, PI3K Signaling, JAK/STAT Signaling, VEGF Signaling, TGF-β Signaling, and likely activation (z-score > 2.0) of PTEN Signaling. Activation of these pathways play a significant role in the development and aggressiveness of various cancers and inhibiting them is an effective treatment for different types of hematological and solid tumors (30, 31). Hence, NSP-B mediated inhibition and activation of these key signaling pathways highlight the anti-leukemic mechanism. Protein-protein interaction (PPI) analysis revealed key hub genes with high connectivity scores, including down-regulation of BUB1B among the top candidates. The BUB1B plays an oncogenic role in a variety of cancers. Increased expression of BUB1B in glioblastoma is associated with tumor proliferation both *in vitro* and *in vivo*. Reduced expression of BUB1B or suppression of its kinase activity resulted in apoptotic cell death in cancer cells (14). In addition, BUB1B expression is associated with poor prognosis in GBM patients (16). BUB1B expression is regulated by FOXM1 transcription factor through direct binding to BUB1B promoter (15). FOXM1 is a master regulator of cell cycle controlling G1/S transition and mitotic progression; however, its mechanism in cell cycle progression is unknown. It has been reported that FOXM1-BUB1B axis is important for the growth and survival of rhabdomyosarcoma cells (15). This corroborates well with our findings where we show that siRNA knockdown or NSP-B mediated inhibition of FOXM1 downregulates BUB1B expression causing suppression of cell proliferation and induction of apoptosis. Down-regulation of key genes and IPA prediction also revealed inhibition of many targets of *FOXM1*, including *BUB1B.* This likely inhibits *FOXM1*, which in turn inhibits cell cycle progression as predicted by IPA. These findings are in agreement with our recent study where we have shown that the NSP-B treatment of leukemic cells caused cell cycle arrest and apoptosis (9). We further found that K562 and U937 cells co-expressed FOXM1 and BUB1B and treatment with NSP-B downregulated the expression of FOXM1 and BUB1B in a dose-dependent manner. NSP-B also downregulated the FOXM1 regulated target molecules, including Aurora kinase A and B. Interestingly, NSP-B treatment suppressed the expression of cell cycle regulated genes CDK4 and CDK6, further supporting our prediction model findings that FOXM1 and BUB1B are linked and together affects the cell cycle progression.

We also found that expression of *BUB1B* is positively correlated with *FOXM1* in all hematological and solid cancers using TCGA data. To see if the high expression of *BUB1B* has any prognostic importance, we investigated the overall survival in AML from TCGA data. The median overall survival in patients exhibiting higher BUB1B expression was ~10 months compared to ~19 months in those exhibiting lower expression profile; however, the association was not statistically significant (HR 1.35, CI 0.88-2.05; logrank p=0.16). We extended the overall survival analysis of *BUB1B* in other solid tumors in TCGA and found that high expression of *BUB1B* is associated with poor overall survival in most cancer types. Cross-talk between survival pathways is gradually emerging as one of the major reasons for drug resistance in cancer treatment. Resistance to chemotherapy, in many instances occurs due to reactivation of the upstream target molecule *via* a negative feedback mechanism (32). Recently it has been shown that targeting of multiple survival pathways with a combination of specific drugs is more effective than treating with a single drug alone in overcoming cancer resistance (25, 33). Therefore, the combinational therapy is advantageous as each drug’s concentration is significantly reduced when administered together, reducing the toxic effects on normal cells. The thiazole antibiotic siomycin also known as thiostrepton is a potent inhibitor of FOXM1 (34). Cotreatment of leukemic cells with subtoxic doses of thiostrepton and NSP-B caused suppression of cell viability. The combination of thiostrepton and NSP-B treatment of K562 and U937 cells further activated caspase-cascades. Indeed, our study demonstrated that NSP-B significantly reduced cell viability and together with thiostrepton exhibited survival-inhibitory effect that is associated with decreased FOXM1 and BUB1B expression in leukemic cells. The activation of the mitochondrial apoptotic pathway is initiated by Bax conformational changes and translocation to the mitochondrial membrane, thereby leading to changes in the mitochondrial membrane potential and, finally activation and cleavage of caspases. Once caspases are activated, there is cleavage of PARP; an essential enzyme that is required for repairing single-stranded breaks in DNA and is a hallmark of cells undergoing apoptosis. Our results go well with these studies as we have also shown that NSP-B treatment of leukemic cells increased Bax, cleaved PARP and p-H2AX expression and downregulated antiapoptotic protein BCl2 together leading to increased apoptosis. Taken together, the present study provides a novel insight into the mechanism of NSP-B mediated apoptosis in leukemic cells and opens a new window for more research.

## Conclusions

The bioinformatic analysis results revealed several novel and promising NSP-B target genes in leukemia cells. Downregulation of ERBB Signaling, EGF Signaling, PI3K Signaling, JAK/STAT Signaling, VEGF Signaling, TGF-β Signaling, and activation of PTEN Signaling were observed in NSP-B treated leukemic cells. *BUB1B* was among the top candidate hub genes and was significantly correlated with *FOXM1* in TCGA data. *In vitro*, we confirmed that NSP-B significantly downregulates the expression of FOXM1 and BUB1B along with other targets such as AURK A, AURK B CDK4, and CDK6. FOXM1 silencing caused downregulation of BUB1B, and AURK A while significantly upregulating the expression of p-H2AX, cleaved caspase 8 and cleaved PARP. We also found synergistic activity between NSP-B and thiostrepton in inhibiting cell proliferation and inducing apoptosis. Further in-depth investigations are warranted to elucidate the potential significance of combination treatment of NSP-B together with FOXM1 targeting agents in preclinical animal models for the successful treatment of leukemia.

## Data Availability Statement

The datasets presented in this study can be found in online repositories. The names of the repository/repositories and accession number(s) can be found in the article/**Supplementary Material**.

## Author Contributions

SK**;** data curation, writing—review and editing, TM; data curation, Analysis, writing—review and editing, GS; data curation, writing—review and editing, AB; writing—review and editing, KP; review and editing, NO; review and editing, CP; review and editing, MH; review and editing, AA; Conceptualization, writing—review and editing, FA; writing—review and editing, TE-E; writing—review and editing, SU; Conceptualization, supervision, writing—original draft preparation. All authors contributed to the article and approved the submitted version.

## Funding

Medical Research Center Grant no; MRC-01-21-301 (SU), Hamad Medical Corporation, Doha Qatar.

## Conflict of Interest

Authors SK, GS, KP, and SU were employed by Hamad Medical Corporation. CP is employed by Mycosynthetix, Inc.

The remaining authors declare that the research was conducted in the absence of any commercial or financial relationships that could be construed as a potential conflict of interest.

## Publisher’s Note

All claims expressed in this article are solely those of the authors and do not necessarily represent those of their affiliated organizations, or those of the publisher, the editors and the reviewers. Any product that may be evaluated in this article, or claim that may be made by its manufacturer, is not guaranteed or endorsed by the publisher.

## Supplementary Material

Supplementary Figure 1 Spearman correlation between BUB1B and FOXM1 in TCGA cancers plotted using log2 TPM expression values. Correlation score (r) and p values (p) are plotted on top left. LAML, Acute Myeloid Leukemia; ACC, Adrenocortical carcinoma; BLCA, Bladder Urothelial Carcinoma; LGG, Brain Lower Grade Glioma; BRCA, Breast invasive carcinoma; CESC, Cervical squamous cell carcinoma and endocervical adenocarcinoma; CHOL, Cholangiocarcinoma; LCML, Chronic Myelogenous Leukemia; COAD, Colon adenocarcinoma; ESCA, Esophageal carcinoma; GBM, Glioblastoma multiforme; HNSC, Head and Neck squamous cell carcinoma; KICH, Kidney Chromophobe; KIRC, Kidney renal clear cell carcinoma; KIRP, Kidney renal papillary cell carcinoma; LIHC, Liver hepatocellular carcinoma; LUAD, Lung adenocarcinoma; LUSC, Lung squamous cell carcinoma; DLBC, Lymphoid Neoplasm Diffuse Large B-cell Lymphoma; MESO, Mesothelioma; MISC, Miscellaneous; OV, Ovarian serous cystadenocarcinoma; PAAD, Pancreatic adenocarcinoma; PCPG, Pheochromocytoma and Paraganglioma; PRAD, Prostate adenocarcinoma; READ, Rectum adenocarcinoma; SARC, Sarcoma; SKCM, Skin Cutaneous Melanoma; STAD, Stomach adenocarcinoma; TGCT, Testicular Germ Cell Tumors; THYM, Thymoma; THCA, Thyroid carcinoma; UCS, Uterine Carcinosarcoma; UCEC, Uterine Corpus Endometrial Carcinoma; UVM, Uveal Melanoma.

Supplementary Figure 2 Overall survival BUB1B for the solid tumors from TCGA. Higher expression of BUB1B is associated with poor overall survival in six different cancers.

Supplementary Figure 3 NSP-B synergistically enhanced the antiproliferative response of thiostrepton in leukemic cells. K562 **(A)** and U937 **(B)** cells were treated with various combinations of NSP-B and thiostrepton for 48 hours and graphs were generated.K562 and U937 cells were treated with different combinations of NSP-B and thiostrepton for 48 hours and cell viability assay were performed as mentioned in the method section. Different types of graphs were prepared using Calcusyn software as described by Chou and Talay.
