# Supplementary figures and images for: Bioinformatics Analysis Reveals FOXM1/BUB1B Signaling Pathway as a Key Target of Neosetophomone B in Human Leukemic Cells: A Gene Network-Based Microarray Analysis
Source: Front Oncol. 2022 Jul 1;12:929996. doi: 10.3389/fonc.2022.929996 (PMC9283897; doi:10.3389/fonc.2022.929996)

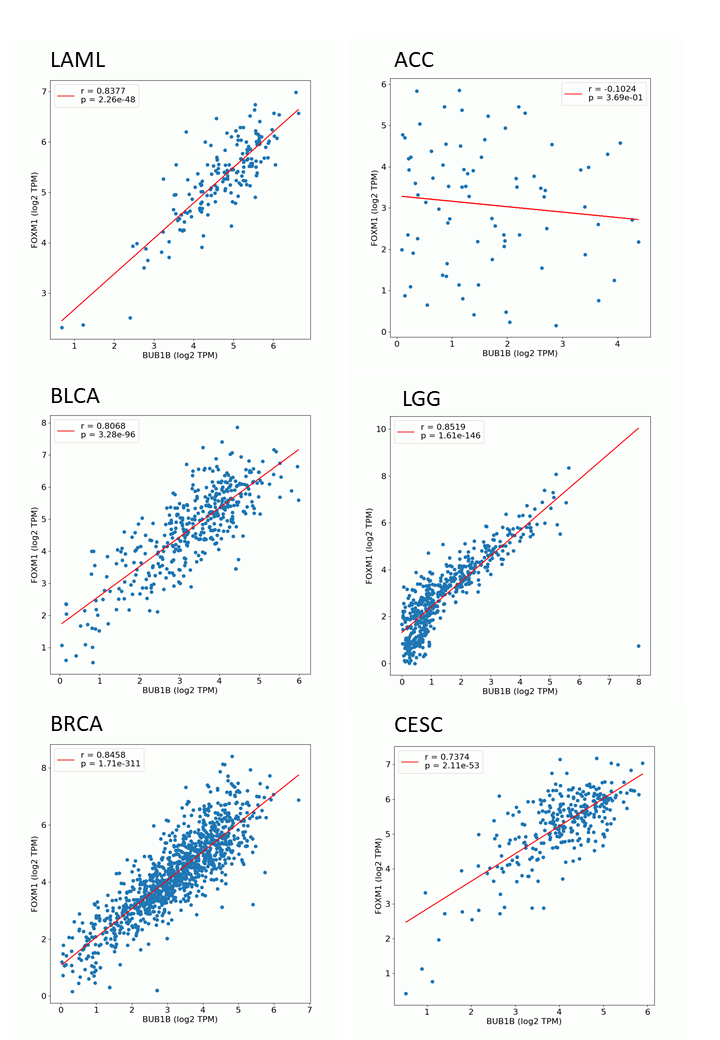

Supplement: Supplementary Figure 1 — Spearman correlation between BUB1B and FOXM1 in TCGA cancers plotted using log2 TPM expression values. Correlation score (r) and p values (p) are plotted on top left. LAML, Acute Myeloid Leukemia; ACC, Adrenocortical carcinoma; BLCA, Bladder Urothelial Carcinoma; LGG, Brain Lower Grade Glioma; BRCA, Breast invasive carcinoma; CESC, Cervical squamous cell carcinoma and endocervical adenocarcinoma; CHOL, Cholangiocarcinoma; LCML, Chronic Myelogenous Leukemia; COAD, Colon adenocarcinoma; ESCA, Esophageal carcinoma; GBM, Glioblastoma multiforme; HNSC, Head and Neck squamous cell carcinoma; KICH, Kidney Chromophobe; KIRC, Kidney renal clear cell carcinoma; KIRP, Kidney renal papillary cell carcinoma; LIHC, Liver hepatocellular carcinoma; LUAD, Lung adenocarcinoma; LUSC, Lung squamous cell carcinoma; DLBC, Lymphoid Neoplasm Diffuse Large B-cell Lymphoma; MESO, Mesothelioma; MISC, Miscellaneous; OV, Ovarian serous cystadenocarcinoma; PAAD, Pancreatic adenocarcinoma; PCPG, Pheochromocytoma and Paraganglioma; PRAD, Prostate adenocarcinoma; READ, Rectum adenocarcinoma; SARC, Sarcoma; SKCM, Skin Cutaneous Melanoma; STAD, Stomach adenocarcinoma; TGCT, Testicular Germ Cell Tumors; THYM, Thymoma; THCA, Thyroid carcinoma; UCS, Uterine Carcinosarcoma; UCEC, Uterine Corpus Endometrial Carcinoma; UVM, Uveal Melanoma. [file Image_1.tif]

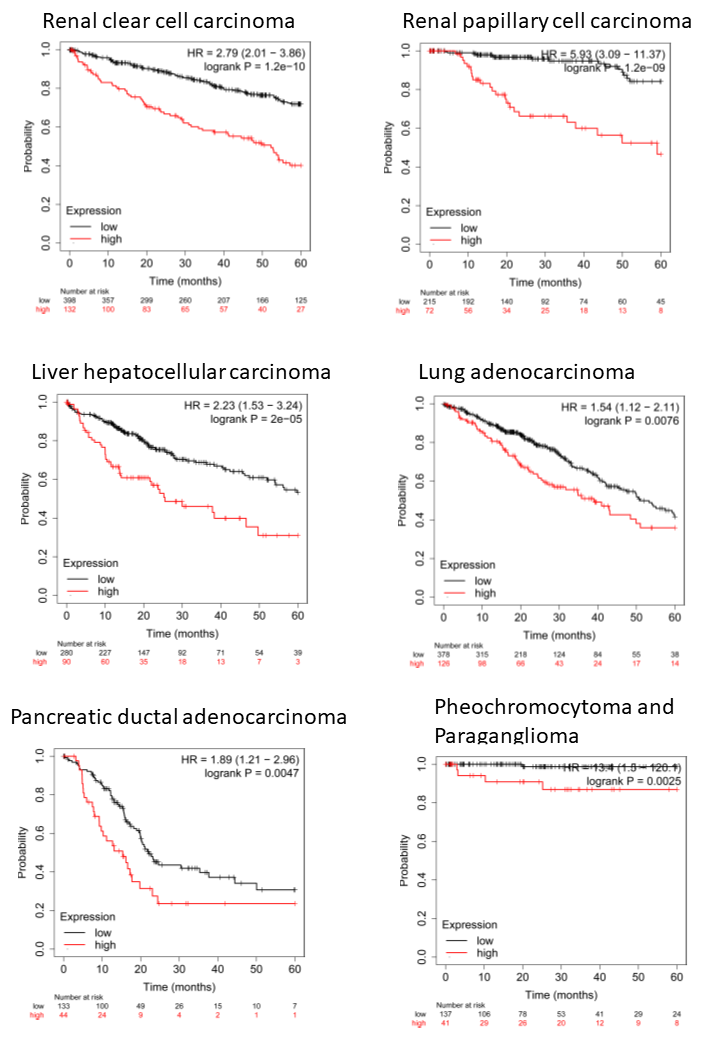

Supplement: Supplementary Figure 2 — Overall survival BUB1B for the solid tumors from TCGA. Higher expression of BUB1B is associated with poor overall survival in six different cancers. [file Image_2.tif]

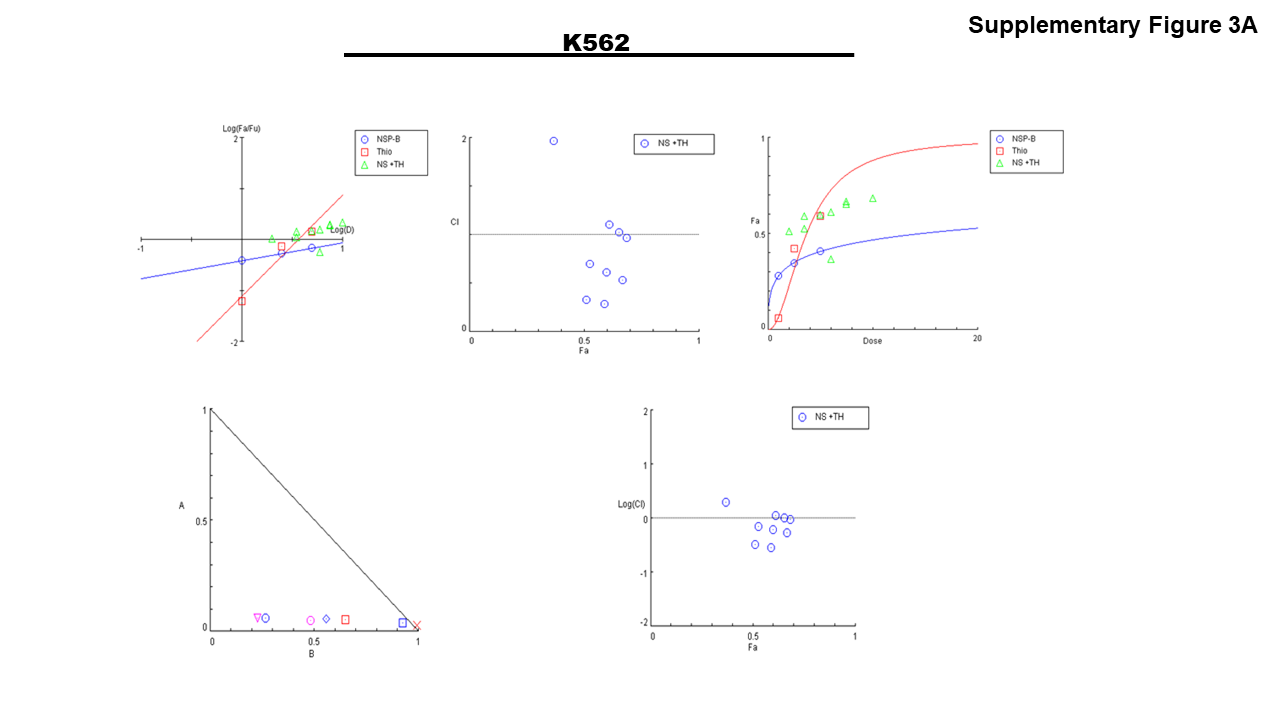

Supplement: Supplementary Figure 3 — NSP-B synergistically enhanced the antiproliferative response of thiostrepton in leukemic cells. K562 (A) and U937 (B) cells were treated with various combinations of NSP-B and thiostrepton for 48 hours and graphs were generated.K562 and U937 cells were treated with different combinations of NSP-B and thiostrepton for 48 hours and cell viability assay were performed as mentioned in the method section. Different types of graphs were prepared using Calcusyn software as described by Chou and Talay. [file Image_3.tif]

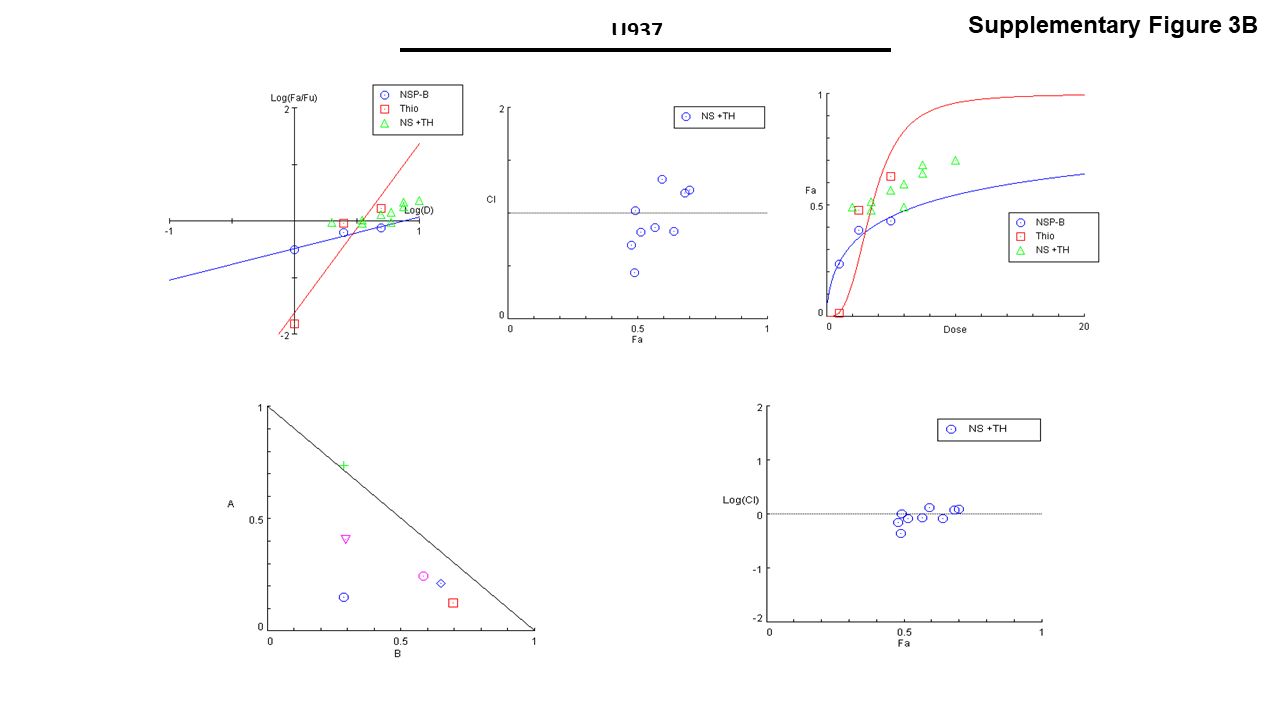

Supplement: Supplementary file 4 [file Image_4.tif]
