# Supplementary material for: Bioinformatics Analysis Reveals FOXM1/BUB1B Signaling Pathway as a Key Target of Neosetophomone B in Human Leukemic Cells: A Gene Network-Based Microarray Analysis
Source: Front Oncol. 2022 Jul 1;12:929996. doi: 10.3389/fonc.2022.929996 (PMC9283897; doi:10.3389/fonc.2022.929996)
Supplement: Supplementary file 6 [file Table_2.docx]

**Table 2: Combination Index calculation using Chou and Talalay method in K562 cell lines:**

**--------------------------------------------------------------------K562 (48hr)--------------------------------------------------------**

| \| **Thio (uM)** \| **NSP-B (uM)** \| **Fractional effect (Fa)** \| **Combination Index (CI)** \| **Dose Reduction Index (DRI)**  **Thio**  **(µM)** \| **Dose Reduction Index (DRI)**  **NSP-B**  **(uM)** \| \| --- \| --- \| --- \| --- \| --- \| --- \| \| **1** \|  \| **0.059** \|  \|  \|  \| \| **2.5** \|  \| **0.423** \|  \|  \|  \| \| **5** \|  \| **0.592** \|  \|  \|  \| |
| --- | --- | --- | --- | --- | --- | --- | --- | --- | --- | --- | --- | --- | --- | --- | --- | --- | --- | --- | --- | --- | --- | --- | --- | --- |

**Median Dose (Dm) = 3.652**

**Exponent shape of curve (m)= 1.99 ±0.477**

**Linear correlation coefficient (r) = 0.972**

| **Thio (uM)** | **NSP-B (uM)** | **Fractional effect (Fa)** | **Combination Index (CI)** | **Dose Reduction Index (DRI)**  **Thio**  **(µM)** | **Dose Reduction Index (DRI)**  **NSP-B**  **(uM)** |
| --- | --- | --- | --- | --- | --- |
|  | **1** | **0.282** |  |  |  |
|  | **2.5** | **0.348** |  |  |  |
|  | **5** | **0.409** |  |  |  |

**Median Dose (Dm) = 14.511**

**Exponent shape of curve (m) = 0.351 ± 0.011**

**Linear correlation coefficient (r) = 0.999**

| **Thio (uM)** | **NSP-B (uM)** | **Fractional effect (Fa)** | **Combination Index (CI)** | **Dose Reduction Index (DRI)**  **Thio**  **(µM)** | **Dose Reduction Index (DRI)**  **NSP-B**  **(uM)** |
| --- | --- | --- | --- | --- | --- |
| **1** | **1** | **0.511** | **0.328** | **3.734** | **16.449** |
| **2.5** | **2.5** | **0.599** | **0.614** | **1.787** | **18.205** |
| **5** | **5** | **0.685** | **0.964** | **1.079** | **26.531** |
