# Supplementary material for: Bioinformatics Analysis Reveals FOXM1/BUB1B Signaling Pathway as a Key Target of Neosetophomone B in Human Leukemic Cells: A Gene Network-Based Microarray Analysis
Source: Front Oncol. 2022 Jul 1;12:929996. doi: 10.3389/fonc.2022.929996 (PMC9283897; doi:10.3389/fonc.2022.929996)
Supplement: Supplementary file 7 [file Table_3.docx]

**Table 3: Combination Index calculation using Chou and Talalay method in U937 cell lines:**

**--------------------------------------------------------------------U937 (48hr)--------------------------------------------------------**

| \| **Thio (uM)** \| **NSP-B (uM)** \| **Fractional effect (Fa)** \| **Combination Index (CI)** \| **Dose Reduction Index (DRI)**  **Thio**  **(µM)** \| **Dose Reduction Index (DRI)**  **NSP-B**  **(uM)** \| \| --- \| --- \| --- \| --- \| --- \| --- \| \| **1** \|  \| **0.015** \|  \|  \|  \| \| **2.5** \|  \| **0.477** \|  \|  \|  \| \| **5** \|  \| **0.629** \|  \|  \|  \| |
| --- | --- | --- | --- | --- | --- | --- | --- | --- | --- | --- | --- | --- | --- | --- | --- | --- | --- | --- | --- | --- | --- | --- | --- | --- |

**Median Dose (Dm) = 3.515**

**Exponent shape of curve (m)= 3.008 ± 1.004**

**Linear correlation coefficient (r) = 0.948**

| **Thio (uM)** | **NSP-B (uM)** | **Fractional effect (Fa)** | **Combination Index (CI)** | **Dose Reduction Index (DRI)**  **Thio**  **(µM)** | **Dose Reduction Index (DRI)**  **NSP-B**  **(uM)** |
| --- | --- | --- | --- | --- | --- |
|  | **1** | **0.237** |  |  |  |
|  | **2.5** | **0.387** |  |  |  |
|  | **5** | **0.43** |  |  |  |

**Median Dose (Dm) = 7.194**

**Exponent shape of curve (m) = 0.562 ± 0.145**

**Linear correlation coefficient (r) = 0.968**

| **Thio (uM)** | **NSP-B (uM)** | **Fractional effect (Fa)** | **Combination Index (CI)** | **Dose Reduction Index (DRI)**  **Thio**  **(µM)** | **Dose Reduction Index (DRI)**  **NSP-B**  **(uM)** |
| --- | --- | --- | --- | --- | --- |
| **1** | **1** | **0.49** | **0.437** | **3.469** | **6.701** |
| **2.5** | **2.5** | **0.568** | **0.862** | **1.54** | **4.679** |
| **5** | **5** | **0.701** | **1.224** | **0.933** | **6.536** |
